# Cell Envelope of Corynebacteria: Structure and Influence on Pathogenicity

**DOI:** 10.1155/2013/935736

**Published:** 2013-01-21

**Authors:** Andreas Burkovski

**Affiliations:** Lehrstuhl für Mikrobiologie, Friedrich-Alexander-Universität Erlangen-Nürnberg, Staudtstr**β**e 5, 91058 Erlangen, Germany

## Abstract

To date the genus *Corynebacterium* comprises 88 species. More than half of these are connected to human and animal infections, with the most prominent member of the pathogenic species being *Corynebacterium diphtheriae*, which is also the type species of the genus. *Corynebacterium* species are characterized by a complex cell wall architecture: the plasma membrane of these bacteria is followed by a peptidoglycan layer, which itself is covalently linked to a polymer of arabinogalactan. Bound to this, an outer layer of mycolic acids is found which is functionally equivalent to the outer membrane of Gram-negative bacteria. As final layer, free polysaccharides, glycolipids, and proteins are found. The composition of the different substructures of the corynebacterial cell envelope and their influence on pathogenicity are discussed in this paper.

## 1. The Genus *Corynebacterium*

The genus *Corynebacterium *belongs to the class of *Actinobacteria* (high G+C Gram-positive bacteria) and comprises a collection of morphologically similar, irregular- or club-shaped nonsporulating (mico)aerobic microorganisms [1, 2]. To date, 88 species were taxonomically classified [3]. More than half of these, that is, 53 species, are occasional or rare causes of infections, with the most prominent member of the pathogenic species being *Corynebacterium diphtheriae*, which is also the type species of the whole genus. Several pathogenic species are considered to be part of the human skin flora, for example, *Corynebacterium amycolatum* or *Corynebacterium jeikeium*, others are considered as zoonotic agents, for example, *Corynebacterium pseudotuberculosis*, *Corynebacterium ulcerans,* or *Corynebacterium xerosis* [3]. Biotechnologically important species used for the industrial production of nucleotides and amino acids are *Corynebacterium ammoniagenes*, *Corynebacterium efficiens,* and *Corynebacterium glutamicum. C. glutamicum* especially is dominating the field of white (bacterial) biotechnology with a production of two million tons of L-glutamate and 1.8 million tons of L-lysine per year [4] and an increasing application as platform organism for the industrial production of various metabolites [5].

Based on their medical importance as etiological agent of diphtheria and their enormous biotechnological potential, *C. diphtheriae* and *C. glutamicum*, respectively, have been the best investigated *Corynebacterium* species for many years. In fact, these were also the first species for which genome sequences became available [6–8]. With the development of high throughput sequencing techniques, to date numerous genome sequences are available including those of *Corynebacterium aurimucosum* [9], *Corynebacterium bovis *[10], various *C. diphtheriae* strains [11–13], *C. jeikeium* [14], *Corynebacterium kroppenstedtii* [15], several *C. pseudotuberculosis *strains [16–24], *Corynebacterium resistens* [25], two *C. ulcerans *strains [26], *Corynebacterium urealyticum* [27], and *Corynebacterium variabile* [28]. This wealth of genome information now provides a basis for the characterization of corynebacterial cell envelope components and corresponding genes both on the whole cell level by global analysis techniques (genomics, transcriptomics, and proteomics) and on a molecular level by specific approaches. 

## 2. General Cell Envelope Architecture

**Figure 1:**
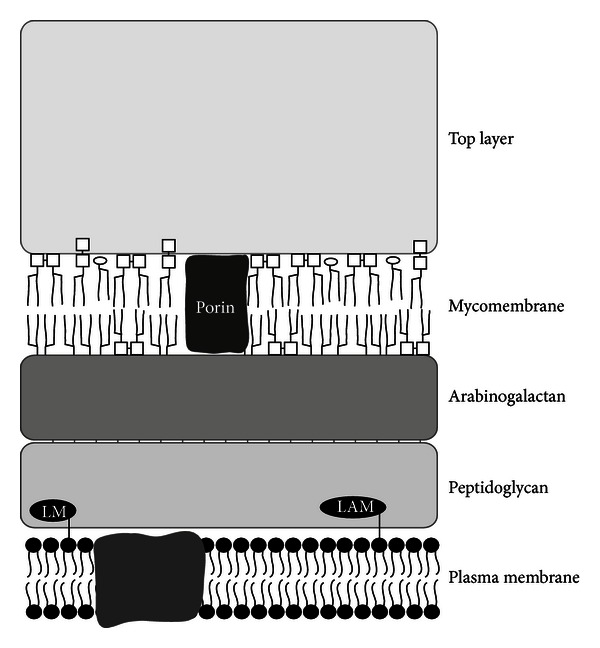
The corynebacterial cell envelope. A schematic representation of the general envelope structure of corynebacteria is shown. The different layers are found in almost all species, and distribution of single components like lipomannan (LM) and lipoarabinomannan (LAM) might differ (for details, see text). Porin proteins might also be present in top layer, S-layer, and pili proteins not shown (figure courtesy of S. Morbach, Friedrich-Alexander-Universität Erlangen-Nürnberg).

Almost all *Corynebacterium* species are characterized by a complex cell wall architecture: the plasma membrane of these bacteria is covered by a peptidoglycan layer, which itself is covalently linked to arabinogalactan, an additional heteropolysaccharide meshwork. Bound to this, an outer layer of mycolic acids is found which is functionally equivalent to the outer membrane of Gram-negative bacteria. As top layer, outer surface material composed of free polysaccharides, glycolipids, and proteins (including S-layer proteins, pili, and other surface proteins) is found (reviewed in [29, 30], see also Figure 1). 

The corynebacterial cell envelope has been investigated by different optical techniques. Electron microscopy of thin sections after freeze substitution revealed a layered cell envelope organization, comprising a plasma membrane, a thick electron-dense layer, an electron-transparent layer, and a thin outer layer [31–33]. This picture resembles the typical mycobacterial cell envelope appearance. The electron-dense layer is traditionally interpreted as peptidoglycan, the electron-transparent layer as mycolic acid layer. When comparing the thickness determined from the electron microscopic pictures of corynebacteria and mycobacteria, the question arose, why their electron-transparent layer has a similar thickness despite the fact that mycobacterial mycolic acids have about a threefold length compared to corynebacterial ones [33]. A solution of this problem was indicated by cryoelectron tomography studies. These revealed a typical outer membrane with a bilayer structure in* Mycobacterium smegmatis*, *Mycobacterium bovis,* and *C. glutamicum*. The thickness of mycobacterial outer layers was smaller than expected and, based on this observation, an alternative model for mycolic acid distribution was proposed, implicating a folding of mycolic acids [34, 35].

The general structure and composition of the corynebacterial cell envelope were earlier reviewed by [29, 30] in respect to biochemical and genetic properties especially in *C. glutamicum*. The aim of this paper will be the presentation of cell envelope properties with a broader focus on different corynebacteria and the importance of cell envelope components for pathogen host interaction.

## 3. Cytoplasmic Membrane and Fatty Acid Synthesis

### 3.1. Plasma Membrane Composition

The cytoplasmic or plasma membrane is the main diffusion barrier of cells and separates cytoplasm and environment. As in other bacteria, the corynebacterial plasma membrane is mainly composed of phospholipids, assembled into a lipid bilayer, which additionally contains other polar lipids besides a great variety of proteins crucial for transport processes and bioenergetics of the cell.

The main phospholipid found in *C. glutamicum* is phosphatidylglycerol, followed by diphosphatidylglycerol, phosphatidylinositol, and minor amounts of phosphatidylinositol dimannosides (PIM_2_) [33, 36–38]. Fatty acids dominating in the plasma membrane are the saturated palmitic acid (16:0) and the desaturated decenoic acid (18:1) [39–42]. However, as in other bacteria, fatty acid composition might change significantly depending on environmental conditions such as low or high temperature [43] or the carbon source available [42].

### 3.2. Fatty Acid Synthesis

Fatty acids are synthesized by successive cycles of multistep reactions [44]. Two distinct types of these fatty acid synthases (FASs) are distinguished based on their general composition: the FAS-II type, characteristically found in bacteria, contains the minimum seven functional domains necessary for fatty acid synthesis organized in one polypeptide, whereas the FAS-I type, typically found in eukaryotes, is comprised of a large multifunctional protein complex. Interestingly and as an exception from the rule FAS-I proteins are found in members of the *Corynebacterineae*. *C. glutamicum,* and *C. efficiens* contain even two FAS-I-type complexes, FAS-IA and FAS-IB, which were functionally characterized in detail for *C. glutamicum *[42]. FAS-IA is essential in *C. glutamicum*, while FAS-IB is not; however, FAS-IB-devoid mutant strains exhibit an altered pattern of fatty and mycolic acids, showing that FAS-IB is active and necessary to generate the typical wild-type fatty acid profile [42]. The regulatory mechanism allowing adaptation of FAS activity to environmental stimuli (see above) is unknown. 

*C. glutamicum* does not only have genes coding for two FAS-I but also genes coding for FAS-II [42], which are involved in elongation of mycolic acid chains in mycobacteria. Since *C. glutamicum* does not contain elongated mycolic acids and FAS-II is absent in other corynebacteria such as *C. diphtheriae*, it was speculated that these proteins might play a minor physiological role or even not be functional in *C. glutamicum *[42]. 

### 3.3. Lipomannan and Lipoarabinomannan

As in other pro- and eukaryotes, the corynebacterial lipid bilayer of the cytoplasmic membrane is not symmetric. In corynebacteria, the proposed reason for asymmetry is an insertion of glycoconjugates in the outer sheet of the cytoplasmic membrane [29]. Lipomannan (LM) and lipoarabinomannan (LAM) derivatives were found in different *Corynebacterium* species [33]. They might be inserted into the plasma membrane via covalently bound palmitic acid or decenoic acid molecules, besides their appearance in the outer surface material of corynebacteria. Distribution of LM- and LAM-like substances seems to be species-specific: in *C. glutamicum* LM-like molecules are dominating, in *C. xerosis* and *C. amycolatum *LAM-like substances were preferentially found, while a *C. diphtheriae* strain showed an almost equal distribution of LM and LAM derivatives [33]. An excellent review dealing with the synthesis of PIM, LM, and LAM derivatives was published recently [45].

### 3.4. Lipoarabinomannan as a Virulence Factor

A 10 kDa lipoarabinomannan polymer was identified in *C. diphtheriae* and designated CdiLAM. The basic structure of CdiLAM shows similarity to mycobacterial LAM; however, in contrast to lipoarabinomannans in other *Actinobacteria*, CdiLAM presents an unusual substitution at position 4 of *α* 1→6 mannan backbone by *α*-*D-*Ara*f*. CdLAM might be part of the plasma membrane but is also located at the surface of *C. diphtheriae* (see the following) and facilitates binding to epithelial cells [46].

The role of lipoarabinomannan with respect to initiation of immune responses was addressed in *C. glutamicum* recently [47]. Characterization of a *C. glutamicum* strain devoid of *α*(1→2) arabinofuranosyltransferase AftE, revealed that AftE is involved in the synthesis of arabinans of LAM. Absence of AftE leads to a hypermannosylated variant of LAM, designated hLM. Both, LAM and hLM were able to modulate the initiation of immune response by interacting with TLR2. As shown by a number of *in vitro* assays, arabinose branching of lipoarabinomannan impacts T-helper-cell differentiation and LAM as well as hLM activate dendritic cells via TLR2. Interestingly, alterations of lipoarabinomannan seem to be discriminated by TLR2 and signal pathway induction by hLM was shown to be broader. In accordance with this observation, hLM was shown to be a stronger inducer of immune responses in mice.

## 4. The Cell Wall Heteropolysaccharide Meshwork

In contrast to, for example, *Escherichia coli* or *Bacillus subtilis*, the cell wall skeleton of corynebacteria and related taxa is not exclusively composed of peptidoglycan, but, in addition, a layer of arabinogalactan is covalently bound to the peptidoglycan, which itself is linked to mycolic acids (for review see [29, 33, 48]). 

### 4.1. Peptidoglycan

As in other bacteria, the glycan part of the murein sacculus is composed of alternating *β*-1,4-linked *N-*acetylglucosamine and N-acetyl muramic acid units, which form the glycan part of the macromolecule. Cross-linking between different glycan polymers occurs via peptide side chains attached to the carboxyl group of muramic acid via peptide bonds. Corynebacterial peptidoglycan is directly cross-linked, as shown for *C. bovis*, *Corynebacterium pseudodiphthericum*, *C. pseudotuberculosis*, *C. striatum*, *C. ulcerans,* and *C. xerosis* [49]. Interpeptide bridges as found in other Gram-positives are absent. In summary, the peptidoglycan of *Corynebacterium in sensu stricto* is of the A1*γ* type [49]. In *C. diphtheriae*, the major peptide units found are the tetrapeptide L-Ala-D-Glu-*meso*-DAP-D-Ala and the tripeptide L-Ala-D-Glu-meso-DAP [50]. Interestingly, only a portion of the peptide side chains are cross-linked via D-Ala-*meso*-DAP bridges, while the others are supposed to be connected by DAP-DAP bridges [29]. 

 Due to the similar peptidoglycan structure, as well as the homology and synteny of genes involved in cell wall synthesis, peptidoglycan synthesis is assumed to be similar to that in *E. coli* [34] (for a topical review on *E. coli* cell wall synthesis, see [51]) and can be separated into three distinct parts. First, the building blocks of peptidoglycan have to be synthesized in the cytoplasm. For this purpose, UDP-N-acetylglucosamine is synthesized and partially converted to UDP-N-acetylmuramic acid by the *murA* and *murB* gene products [30, 52]. Next, UDP-N-acetylmuramyl-pentapeptide is formed, a process in which the *murE*, *murF*, *murD,* and *murC* gene products are involved. 

The second step of building block synthesis is located at the cytoplasmic membrane and involves transfer of a phospho-N-acetylmuramyl-pentapeptide to polyprenol phosphate catalyzed by the *mraY* gene product and resulting in lipid I. As in many other bacteria, undecaprenol (C_55_) might be the polyprenol used, since at least in *C. glutamicum* this compound is used for polyprenyl monophosphomannose synthesis [53]. Next, N-acetylglucosamine is transferred from UDP-N-acetylglucosamine to lipid I. This is catalyzed by *murG* and yields lipid II or N-acetylglucosamine-*β*-(1,4)-N-acetylmuramyl(pentapeptide)-pyrophosphoryl-polyprenol. The generated lipid intermediate mediates the transport of the hydrophilic disaccharide pentapeptide precursor from the cytoplasm across the hydrophobic plasma membrane to the peptidoglycan layer. 

As third step, disaccharide pentapeptide precursors are integrated into the growing peptidoglycan meshwork by transglycosylation and transpeptidation reactions catalyzed by penicillin binding proteins [54]. Typically, several copies of these can be found in different *Corynebacterium* species such as *C. diphtheriae* [52] and *C. glutamicum* [55] (for review, see [30]). For *C. glutamicum* five out of nine putative penicillin binding proteins were shown to be functional in peptidoglycan synthesis [55]. 

A strict coordination of the described steps of cell wall synthesis is crucial for survival of bacteria, since otherwise the cells would be prone to disruption due to the high internal turgor pressure. The regulation of this process and cell division has been reviewed for *C. glutamicum* [54] and other *Actinobacteria* [56] recently. 

### 4.2. Linker Unit

As shown in mycobacteria, arabinogalactan is covalently bound to the murein sacculus via a polysaccharide linker unit making up phosphodiester bonds to about 10% of the muramic acid residues of the peptidoglycan [57]. The mycobacterial linker unit consists of galactose, rhamnose, and N-acetylglucosamine linked as Gal*f*-(1→4)-Rha*p*-(1→3)-GlcNAc via a 1-*O*-phosphoryl bond of GlcNAc to the 6-OH position of muramic acid [58]. Studies of *C. diphtheriae* revealed a very similar linkage profile of arabinogalactan to that of *Mycobacterium tuberculosis* [30, 33]. 

### 4.3. Arabinogalactan

Arabinogalactan is a heteropolysaccharide consisting of D-arabinose and D-galactose present in their furanose form (Ara*f*, Gal*f*). In *C. glutamicum* alternating *β*(1→5) and *β*(1→6)-linked Gal*f* residues form a linear chain of up to 30 sugar moieties, which is bound to peptidoglycan by the linker unit. The galactan chain is decorated with arabinan, branched from the C5 of *β*(1→6) linked Gal*f*. In *C. glutamicum* three branched arabinan domains are linked per galactan domain at the 8th, 10th, and 12th Gal*f* residues. Enzymes involved in arabinogalactan synthesis are well characterized in *C. glutamicum* [59–62] and have been reviewed recently [30].

Whereas *C. glutamicum* arabinogalactan consists exclusively of arabinose and galactose, *C. diphtheriae* arabinogalactan contains significant amounts of mannose, and *C. amycolatum* and *C. xerosis* arabinogalactans are characterized by additional glucose content [33]. In any case, arabinogalactan provides a covalent connection not only to peptidoglycan but also to the outer membrane layer.

## 5. Mycolic Acids and Trehalosyl Mycolates

### 5.1. Composition and Biosynthesis

A second permeability barrier equivalent to the outer membrane of Gram-negative bacteria is a key feature of the CMN group (*Corynebacterium*, *Mycobacterium*, and *Nocardia*) of *Actinobacteria*. The functionality of this barrier is critically influenced by its mycolic acid content [63]. The inner half of the corynebacterial mycolic acid layer is mainly formed by mycolic acids esterified to the 5-OH group of the penultimate *β*(1→2)-linked or ultimate Ara*f* residue of arabinogalactan, while in the outer sheet, trehalose and glycerol esterified mycolic acids are predominating. Additionally, minor amounts of free mycolic acids are found. A recent biochemical disclosure of the outer membrane of *C. glutamicum *showed that the lipids composing the mycomembrane consist almost exclusively of mycolic acids derivatives, whereas only minor amounts, if any, of phospholipids and lipomannans were detected [64].

In corynebacteria, fatty acids with around 30 carbon atoms (corynomycolates), in nocardia with about 50 carbon atoms (nocardomycolates), and in mycobacteria with about 70 to ninety carbon atoms (eumycolates) are found [29]. In contrast to the linear fatty acids of the phospholipids, mycolic acids are *α*-branched *β*-hydroxy fatty acids, requiring carboxylation and condensation of two fatty acids for their synthesis [65]. The enzymes involved in these steps were identified by mutation analyses in *C. glutamicum*, and two carboxylases were identified to be essential for mycolic (and fatty) acid synthesis, AccD2 and AccD3 [66]. These are conserved in *Corynebacterineae* and provide the crucial carboxylated intermediate for condensation of merochain and *α*-branch [67]. Further proteins involved in mycolic acid synthesis in *C. glutamicum* and related organisms are AccD1, involved in malonyl-CoA synthesis, Pks, a ketoacyl synthase involved in fatty acid elongation, and FadD, a fatty acid acyl-AMP ligase ([65, 67]; for a recent review see [30]). 

Three pathways for trehalose synthesis were described in *C. glutamicum*: the OtsA-OtsB pathway synthesizing trehalose from UDP-glucose and glucose-6-phosphate, the TreY-TreZ pathway using malto-oligosaccharides or *α*-1,4-glucans as substrate, and the TreS pathway using maltose as an educt for trehalose synthesis [68, 69]. It is suggested that the transfer of mycolic acid moieties to trehalose occurs outside the cytoplasm [70], since in absence of internally synthesized trehalose, either trehalose or externally added glucose, maltose and maltotriose, can be used as substrate for mycolic acid modification and result in the corresponding di- and monocorynomycolates [70].

The production of arabinogalactan-linked mycolates, trehalosylmonocorynomycolates, and trehalosyldicorynomycolates indicates the presence of mycolyltransferases. In fact, proteins similar to the mycobacterial antigen 85 showing mycoloyltransferase activity, also designated fibronectin-binding protein, were identified in corynebacteria. The first member was the PS1 protein from *C. glutamicum *[71]. Later it was shown that six of these proteins are present in this species, five in *C. efficiens,* and four in *C. diphtheriae* [29, 72, 73]. The enzymes are fully redundant in *C. glutamicum* in respect to mycoloyl moiety transfer to trehalose and partially redundant with respect to transfer of arabinogalactan [29, 72, 74]. The proteins associated with mycolic acid transport across the plasma membrane were characterized in *M. smegmatis* and *C. glutamicum* recently [75]. In *C. glutamicum*, four *mmpL* genes encode large membrane proteins associated with mycolate metabolism and transport, which have partially redundant function [75].

Besides the plasma membrane lipids, the outer membrane fatty acid composition also has to be adapted to different temperatures, a crucial process for function of the mycolic acid layer as a diffusion barrier [76]. In fact, one stress-induced protein, designated ElrF, was identified, which is conserved in *Corynebacterineae* and plays a role in the regulation of outer membrane lipid composition in response to heat stress [77].

### 5.2. Corynomycolates and Pathogenicity

Almost all *Corynebacterium* and *Mycobacterium* species are characterized by a complex cell wall architecture comprising an outer layer of mycolic acids, which is functionally equivalent to the outer membrane of Gram-negative bacteria, not only in respect to its physiological role as a permeability barrier, but also as important component of host pathogen interaction. It has long been known that constituents of the mycolic acid layer may be immune stimulatory and effecting macrophage function. These effects are best investigated in *M. tuberculosis* [78], where trehalose dimycolate, also designated as cord factor, inhibits fusion events inside the host macrophage and at the same time contributes to macrophage activation. However, limited data for corynebacteria are available. Investigations of *C. pseudotuberculosis* (formerly *Corynebacterium ovis*) indicate a lethal effect of outer membrane lipids on caprine and murine macrophages. Lipid extracts of *C. pseudotuberculosis* had negative effects on glycolytic activity, viability, and membrane integrity [79]. When macrophages were infected with *C. pseudotuberculosis*, uptake of the bacteria and lysosome fusion was functional, but bacteria survived internalization. However, the macrophages were destroyed [80]. For *C. glutamicum* priming and activation of murine macrophages by trehalose dimycolates were reported [81].

## 6. Top Layer

Cell surface molecules extracted from different corynebacteria consist of over 90% carbohydrates and of a minor portion (less than 10%) of proteins. Analyses of surface saccharides of *C. diphtheriae* strains indicated the presence of sugars such as *N*-acetylglucosamine, *N*-acetylgalactosamine, galactose, mannose, and sialic acid [82]. In *C. amycolatum* and *C. xerosis*, a neutral glucan consisting mainly of glucose with an apparent mass of 110 kDa was found, in addition to arabinomannans consisting of arabinose and mannose in a 1 : 1 ratio of 13 and 1.7 kDa [29, 33]. Additionally, the above-mentioned lipoarabinomannan and lipomannan were detected in the outer layer, besides trehalose dicorynomycolate, trehalose monocorynomycolate, and phospholipids. Interestingly, the same lipid composition was found for whole bacteria, indicating that all classes of lipid molecules were exposed on the corynebacterial cell surface [33], in contrast to the mycobacterial situation [83]. Also, a more distinct composition of plasma membrane and mycomembrane was reported for *C. glutamicum* [64]. 

While Puech and coworkers could only detect a few bands on coomassie-stained SDS polyacrylamide gels for different corynebacteria, proteome analyses supported the idea that a plethora of proteins is exposed on the surface of corynebacteria. Corresponding studies carried out for *C. diphtheriae* [84], *C. efficiens* [85], *C. glutamicum* [64, 85, 86], *C. jeikeium* [87], and *C. pseudotuberculosis* [88] revealed a significant number of proteins for every single species. Often, these proteins are uncharacterized; some clearly have functions for nutrient uptake and growth, while others are involved in host pathogen interactions.

### 6.1. Corynebacterial Porins

Since the permeability barrier of the mycolic acid layer most likely hinders nutrient uptake by plasma membrane transporters, the outer membrane has to be selectively permeabilized to allow growth. For this purpose, porin proteins are inserted into the membrane. In general these are functionally equivalent to Gram-negative porins but resemble a completely different structure, multimers of *α*-helical subunits instead of the Gram-negative trimeric *β*-barrels.

 Corynebacterial porins are best investigated in *C. glutamicum*, where several different channel-forming proteins were identified, namely, PorA, PorB, PorC, and PorH [89–92]. Homologs of these were found in* Corynebacterium callunae*, *C. efficiens* [93], and *C. diphtheria *[94]. Despite the fact that *C. amycolatum* does not have corynemycolic acids and contains only small amounts of extractable lipids [33, 95, 96], a channel-forming protein was also isolated from this pathogenic species [97]. Reconstitution experiments with purified PorA and PorH from *C. glutamicum* and homology studies indicated that the major cell wall channels of *C. callunae*, *C. diphtheriae*, *C. efficiens,* and *C. glutamicum* are formed of two porins, one of the PorA and one of the PorH type [98]. Furthermore, for *C. glutamicum* PorA and PorH, an *O*-mycolation was shown, an extremely unusual modification [99].

Comparative studies of *C. glutamicum *wild type and a PorA-lacking mutant strain revealed a drastically decreased susceptibility of the mutant towards ampicillin, kanamycin, streptomycin, tetracycline, and gentamicin [92]. Corresponding studies with pathogenic corynebacteria are missing; however, based on the structural and functional similarities depicted above, differences in the porin repertoire might be one reason for different antibiotic susceptibility observed for the different species.

### 6.2. S-Layer Proteins

In some *Corynebacterium* strains, the cell surface is covered with a crystalline surface layer composed of single protein species, which are anchored in the corynebacterial outer membrane [100]. In *C. glutamicum*, the S-layer protein PS2 has an apparent molecular weight of 63 kDa and is anchored in the mycomembrane by a C-terminal hydrophobic domain [101]. Electron and atomic force microscopy applications revealed a highly ordered hexagonal S-layer [31, 101–104] and a certain degree of variability in different *C. glutamicum* isolates [103]. The PS2 encoding *cspB* gene is located on a genomic island [103], a situation often found for virulence genes; however, no distinct phenotype was found for S-layer mutant strains.

### 6.3. Sialidase

Sialidases, also designated as neuraminidases, are glycosyl hydrolases that catalyze the removal of terminal sialic acid residues from a variety of glycoconjugates of the host surface [105]. The sugar is subsequently metabolized or used to decorate the surface of the pathogen. In fact, *C. diphtheriae* exposes sialic acids on its outer surface [82]. Sialidase activity was first identified in a crude preparation of diphtheria toxin [106], and sialidase production and composition of cell surface carbohydrates of *C. diphtheriae* seem to be directly depending on iron concentration in the medium [107–109]. A putative exosialidase, designated NanH, was identified in* C. diphtheriae*. Biochemical studies revealed trans-sialylation activity; however, it is still unclear if NanH is involved in sialic acids decoration or not [107]. 

### 6.4. Pili

Proteinaceous protrusions such as fimbriae and pili are pivotal players for the attachment of bacteria to abiotic and biotic surfaces. In corynebacteria, pili were first reported in *Corynebacterium renale* [110]. Later detailed molecular biological analyses were carried out in *C. diphtheriae *(for review, see [111]). *C. diphtheriae* type strain NCTC13129 produces three distinct pilus structures, SpaA-, SpaD-, and SpaH-type pili, which are polymerized by specific class C sortases [112] and covalently linked to cell surface by sortase F [113]. Each type of pilus is composed of the name-giving shaft proteins, SpaA, SpaD, and SpaH, and minor pili subunits, that is, SpaB, SpaC, SpaE, SpaF, SpaG, and SpaI. For SpaB and SpaC, a function in cell line specificity of pathogen attachment was shown [114]. Ott and coworkers observed that different *C. diphtheriae* isolates are characterized by a different pili repertoire [115] which is characterized by different biophysical properties [116]. Interestingly, pili formation and adhesion rate are not strictly coupled processes and bald strains are also able to attach to host cells [115], indicating the presence of adhesion factors besides pili.

 Besides in *C. diphtheriae*, pilus gene clusters were found in several pathogenic corynebacteria including *Corynebacterium accolens*, *C. amycolatum*, *C. aurimucosum*, *Corynebacterium glucuronolyticum*, *C. jeikeium*, *Corynebacterium pseudogenitalium*, *C. striatum*, *Corynebacterium tuberculostearicum,* and *C. urealyticum,* [111].

### 6.5. 67-72p Hemagglutinin

Besides pili, *C. diphtheriae* exhibits nonfimbrial surface proteins, 67-72p [117]. Recent studies indicated that 67-72p is encoded by a single gene, *DIP0733 *[118]. The corresponding gene product, DIP0733, enables *C. diphtheriae* to recognize and bind specifically to human erythrocytes, a process-designated hemagglutination. *In vitro* experiments with protein-coated latex beads indicated that DIP0733 contributes to invasion and induction of apoptosis in HEp-2 cells [118]. 

### 6.6. NlpC/P60 Proteins

Pathogen factors responsible for adhesion are at least partially characterized; however, the molecular background of invasion remains unclear. Based on a comprehensive analysis of proteins secreted by *C. diphtheriae* [84], Ott and coworker [119] started to characterize the surface-associated protein DIP1281, annotated as invasion associated protein. DIP1281 I is a member of the NlpC/P60 family, a large superfamily of several diverse groups of proteins [120], including putative proteases and probably invasion-associated proteins. They are found in bacteria, bacteriophages, RNA viruses, and eukaryotes and various members are highly conserved among nonpathogenic and pathogenic corynebacteria such as *C. diphtheriae*, *C. efficiens*, *C. glutamicum,* and *C. jeikeium*. DIP1281 mutant cells completely lacked the ability to adhere to host cells and consequently to invade these. Based on proteome, fluorescence and atomic force microscopy, it was concluded that DIP1281 is a pleotropic effector of the *C. diphtheriae* outer surface rather than a specific virulence factor [119], an idea that was supported by results obtained for corresponding mutants of the nonpathogenic *C. glutamicum* R strain [121]. Nevertheless, proteins of this family seem to play a major role in corynebacterial cell surface organization and cell separation. Furthermore, the results obtained with *C. diphtheriae* mutant strains [119] support the idea that the corynebacterial cell envelope components are important determinants of host pathogen interactions.

## 7. Concluding Remarks

Corynebacteria show a fascinating complex cell wall synthesis and architecture (see also Figure 1). Enormous progress was made in the characterization of fatty acids, sugar moieties, and cell wall polysaccharides; however, the protein content of the top envelope layer has only been characterized partly, despite its probable importance for intercellular communication and host pathogen interaction, as targets for therapy or its importance for biotechnological production. Furthermore, porin and S-layer proteins may have interesting biotechnological functions in respect to surface display of proteins and setup of nanostructures [100, 122].
